# Accelerating Gut Microbiome Research with Robust Sample Collection

**Published:** 2023-04-03

**Authors:** Zoe J. Zreloff, Danielle Lange, Suzanne D. Vernon, Martha R. Carlin, Raul J. Cano

**Affiliations:** The BioCollective, 5650 Washington Street, Unit C9, Denver CO 80216

**Keywords:** Stool, Fecal, Microbiome, Microbiota, Heterogeneous, Homogeneous, Sample

## Abstract

**Background:**

Inferior quality of biological material compromises data, slows discovery, and wastes research funds. The gut microbiome plays a critical role in human health and disease, yet little attention has been given to optimizing collection and processing methods of human stool.

**Methods:**

We collected the entire bowel movement from 2 healthy volunteers: one to examine stool sample heterogeneity and one to test stool sample handling parameters. Sequencing and bioinformatic analyses were used to examine the microbiome composition.

**Results:**

The microbiome profile varied depending on where the subsample was obtained from the stool. The exterior cortex of the stool was rich in specific phyla and deficient in others while the interior core of the stool revealed opposite microbiome profiles. Sample processing also resulted in varying microbiome profiles. Homogenization and stabilization at 4°C gave superior microbial diversity profiles compared to the fresh or frozen subsamples of the same stool sample. Bacterial proliferation continued in the fresh subsample when processed at ambient temperature. *Bacteroidetes* proliferated and *Firmicutes* diminished during the 30-minute processing of fresh sample. The frozen sample had good overall diversity but Proteobacteria diminished likely because of the freeze/thaw.

**Conclusion:**

The microbiome profile is specific to the section of the stool being sampled. Stool sample collection, homogenization and stabilization at 4°C for 24 hours provides a neat, high-quality sample of sufficient quantity that can be banked into aliquots with nearly identical microbial diversity profiles. This collection pipeline is essential to accelerate our understanding of the gut microbiome in health and disease.

## INTRODUCTION

The more than 10 trillion microbial inhabitants of the gut are a valuable window into health and disease because of the myriad of interactions and influences these organisms have in our bodies. Accordingly, a representative and high-quality sample of the fecal microbiota is essential to advance gut microbiome research. Biological sample collection is the first and most important step in any research and development pipeline. If research testing begins with a sample that was not collected, processed or stored properly and is compromised in any way, the resulting data is unreliable and can lead further research astray or derail it completely.

Microbiome research has increased dramatically and is driven by advances in technology and decreases in sequencing costs. The majority of this research hinges on scientists access to sufficient quantity of high-quality stool samples. While advanced sequencing technologies and sophisticated bioinformatics are used to decipher the human gut microbiota, little attention is paid to methods of collection, processing and storing of the stool sample that go into these sophisticated discovery pipelines. For example, two of the largest microbiome projects, American Gut Project and the Human Microbiome project used different fecal matter collection methods. Participants in the American Gut Project collected their fecal sample in the privacy of their home using a swab that was returned to the laboratory for sequencing *via* the United States Postal Service at ambient temperature ^[1]^. The National Institutes of Health Human Microbiome Project had participants collect their stool sample in a plastic container which was then stored in a Styrofoam container with several frozen gel packs and returned within 24 hours of their bowel movement ^[2]^. Other methods for collecting human stool for microbiome analysis involve using swabs ^[3–5]^, toilet paper wipes ^[6,7]^, scoops ^[8]^, and containers to collect whole stools which can then be used in toto or subsampled with scoops or swabs ^[9,10]^, Comparison of these sampling methods have shown disparity in microbiome composition ^[11,12]^. Standardization of sample collection was identified as one of the key knowledge gaps in microbiome research ^[11,13–15]^.

Optimizing collection, processing, storage and preservation of human stool that is representative of the gut microbiome is essential for biomarker discovery. The objective of this study was to assess heterogeneity of the human stool and optimize collection and homogenization so that the microbiota remained viable and as representative as a recently evacuated stool sample for optimal use in many different omics platforms.

## MATERIALS AND METHODS

### Sample collection

**Figure 1.**
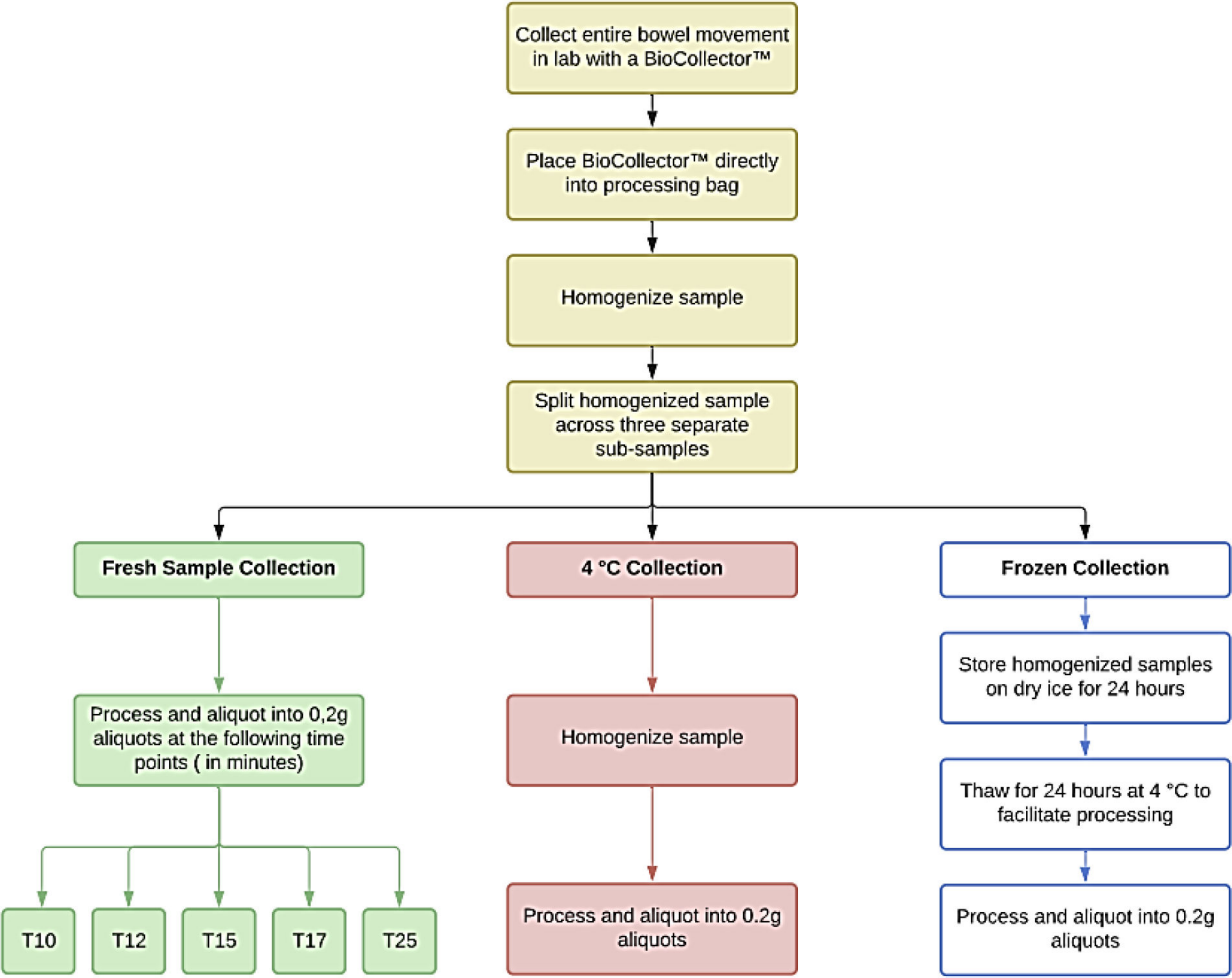
Sample Processing Workflow. This figure shows the steps taken in sample collection and processing for comparing sample preparation protocols.

The entire bowel movement was collected at the laboratory from two healthy volunteers using the BioCollector^™^ according to our IRB-approved protocol (IRB Tracking Number: 20160838). Both stools were Type 4 on Bristol Stool Form Scale ^[16]^. To evaluate sample heterogeneity, the stool from one volunteer was dissected as shown in Figure 1. Samples weighing approximately one gram were taken at four different locations, one cm apart, along the length of the stool. Three sub-sections were each manually homogenized, aliquoted into two replicate aliquots and stored at −80°C. The core of the fourth one-gram sample was separated from the cortex and each was independently homogenized, aliquoted and stored. The remaining material from the stool was manually homogenized and aliquoted. DNA was extracted from the two replicate aliquots from each subsection of the dissected stool for sequencing (see below). To evaluate collection and processing, the stool from the second volunteer was emptied from the BioCollector^™^ into a plastic bag, closed and then thoroughly homogenized for 2 minutes by smashing and scraping using a plastic scraper. No homogenization buffer was used in this process. The homogenized, neat (nothing added) sample was divided into 3 equal subsamples for processing as follows: fresh (fresh); 4°C (4C), and frozen on dry ice (frozen) (Figure 1). The 4C and frozen subsamples were handled first as follows; the 4C subsample was put into a mylar bag and on top of a frozen freezer brick in a Styrofoam container and stored for 24 hours. The frozen subsample was put into a mylar bag and placed in a Styrofoam container with dry ice for 24 hours. The fresh subsample was then processed over approximately 30 minutes into 80 cryovials containing 0.2 grams and frozen at −80°C until sequencing. After 24 hours, the 4C subsample was aliquoted at room temperature into 20 cryovials each with 0.2 grams homogenized material and frozen at −80°C until sequencing. The frozen subsample was thawed at 4°C for 24 hours and then aliquoted at room temperature into 20 cryovials each with 0.2 grams homogenized material and frozen at −80°C until sequencing.

### Library prep and sequencing

DNA from fecal samples was isolated using the QIAGEN DNeasy PowerSoil Pro Kit, according to the manufacturer’s protocol. Isolated DNA was quantified by Qubit (ThermoFisher). A homogenized fecal reference material was included in all library preparations and sequencing runs. DNA libraries for whole genome sequencing were prepared using the illumina Nextera XT library preparation kit, with a modified protocol. Library quantity was assessed with Qubit (ThermoFisher). Libraries were then sequenced on an illumina HiSeq platform 2 × 150 bp. The optimized 16S sequencing covers the V3-V4 (341 nt-805 nt) region of the 16S rRNA gene with a two-step PCR strategy. The first step used the 16S-optimized primer set to amplify the V3-V4 regions of 16S rDNA within the metagenomic DNA. The primer set contained optimized primers for comprehensive taxa coverage and frame shift primers for higher complexity. In addition to specific V3-V4 priming regions, these primers have sequences partially complementary to illumina adapters. The first PCR amplifications were carried out in a 25 ul volume. Each reaction mixture contained 2.5 μl 1X primer mix, 5 ng-50 ng metagenomic DNA, 0.5 μl AccuPrime Taq DNA Polymerase (ThermoFisher) and 2.5 10X AccuPrime PCR Buffer II (ThermoFisher). The PCR conditions included an initial denaturation step at 95°C for 2 minutes, followed by 10 cycles of 95°C for 45 seconds, 57°C for 90 seconds, 72°C for 50 seconds, and end with an extension step at 72°C for 10 minutes. Next, the PCR products from the previous two reactions were mixed at equal amounts and used as templates in the second step to produce illumina dual-index libraries for sequencing, with both adapters containing an 8 bp index allowing for multiplexing. Each reaction mixture contained 0.5 μl AccuPrime Taq DNA Polymerase (ThermoFisher) and 2.5 10X AccuPrime PCR Buffer II (ThermoFisher), 3 μl 10 μM adapter primer D50X, 3 μl 10 μM adapter primer D70X, 4 μl 10 μM Illumina primer cocktail and 50 μl PCR product from first PCR reaction mix. The PCR conditions included an initial denaturation step at 95°C for 2 minutes, followed by 6 cycles of 95°C for 45 seconds, 60°C for 30 seconds, 72°C for 50 seconds, and end with an extension step at 72°C for 10 minutes. The dual-indexed library amplification products were purified using Ampure beads (Beckman Coulter). Library quantification was performed using Qubit dsDNA HS assay (ThermoFisher) and qualified on a 2100 Bioanalyzer instrument (Agilent) to show a distribution with a peak in the expected range. A final qPCR quantification was performed before loading onto an MiSeq (illumina) sequencer for PE250 (v2 chemistry). FastQC analysis of forward and reverse reads was conducted on all raw sequence data prior to use in analytic pipelines to determine the overall quality of the product and as a milestone prior to further analysis and to determine trim parameters for DADA2 ^[17]^. The DADA2 script in QIIME2 ^[18]^ was executed for sequence quality control and feature table construction. This script removes and/or corrects reads with sequencing errors and removes chimeric.

### Bioinformatic analysis

All bioinformatics analysis was conducted using a QIIME2 version 2020.2 workflow similar to that described in QIIME2 for analyzing “Moving Pictures” data (https://docs.qiime2.org/2020.2/tutorials/moving-pictures/). Paired-end sequencing reads were imported into the workflow using Casava v1.8.2 d. Sequence quality control and features table construction was conducted using DADA2 ^[17]^. Taxonomic analysis was conducted using the Silva ^[19]^ 132 99% OTUs, full length, seven level taxonomy classifier (silva-132-99-nb-classifier.qza).

### Quality assurance

Two aliquots of a reference material derived from homogenized whole stools and fully characterized, both by metagenomics and metabolomic analysis were included, blindly, with the test fecal samples for process quality control. FastQC analysis of forward and reverse reads was conducted on all raw sequence data prior to use in analytic pipelines (https://www.bioinformatics.babraham.ac.uk/projects/fastqc/) to determine the overall quality of the product and as a milestone prior to further analysis and determine trim parameters for DADA2 ^[17]^. The DADA2 script in QIIME2 ^[18]^ was executed for sequence quality control and feature table construction. This script removes and/or corrects reads with sequencing errors and removes chimeric.

### Statistical analyses

All statistical analyses were performed using packages ‘vegan’ v2.5–6 and ‘ggplot2’ v3.3.2 in R 3.6.3 (https://www.r-project.org/). For microbiome analysis, rarefaction depth was set at 25000 reads. Shannon diversity index ^[20,21]^. Chao1 Index ^[21]^ and Pielou’s Evenness ^[22]^ were used to evaluate alpha (within sample) diversity. Beta (between sample) diversity was examined using multidimensional scaling analysis (MDA) ^[23]^ of Bray-Curtis ^[24]^ and Jaccard ^[25]^ distances. The Wilcoxon rank-sum test ^[26]^ was used to compare alpha diversity values between groups (p>0.05). Statistical significance of beta-diversity distances between stool processing workflows was assessed using PERMANOVA ^[27]^ with 999 permutations. Alpha diversity group significance was calculated using nonparametric Kruskal-Wallis H test ^[28]^.

## RESULTS

Two aliquots of a reference material derived from homogenized whole stools were included in the batch submitted for sequencing to the test fecal samples for process quality control. Sequence count for both forward and reverse reads were 251 bp. The mean Q value for forward reads was 36 while that of the reverse reads was 34. Based on these data the sequences were trimmed to 200 bp prior to DADA2 analysis ^[29]^. The average number of input-reads for DADA2 processing was 75132 ± 6052, of which 37175 ± 5556 passes denoising and chimera analysis (Table S1). The “blind” reference sample passed QC based on taxonomic profile (Figure S1), Wilcoxson Rank Sum Test (P-value=0.3173) and Permutational analysis of variance (PERMANOVA) (statistic=0.989, P-value=0.532) ^[30,31]^. To ascertain heterogeneity, the stool sample from one volunteer was dissected as shown in Figure 1. Sequencing depth was 57720 ± 23466.

**Figure 2.**
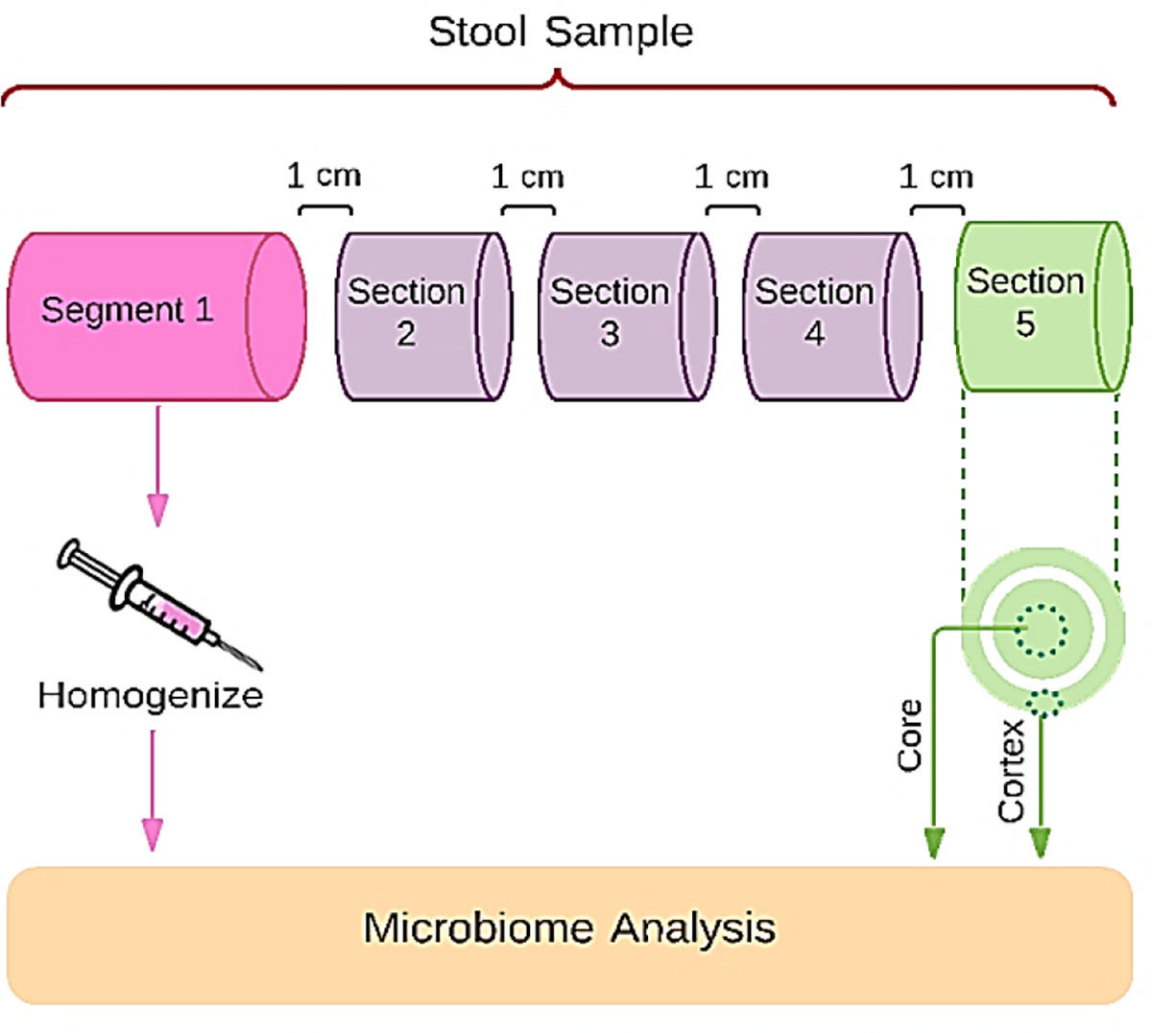
Schematic representation of the stool dissection study to evaluate the level of microbiome homogeneity of each section. Each section was processed independently and analysed for microbiome composition.

Each section of the stool had dissimilar microbial composition (Figure 2). For example, section 1 was significantly dissimilar (p<0.01) from both the core and cortex of section 6. There were differences in microbiome composition of each section. Subsamples Replicate aliquots from sections 1,3, and cortex were all dissimilar from each other while the Segment 1 replicate aliquots were similar.

**Figure 3.**
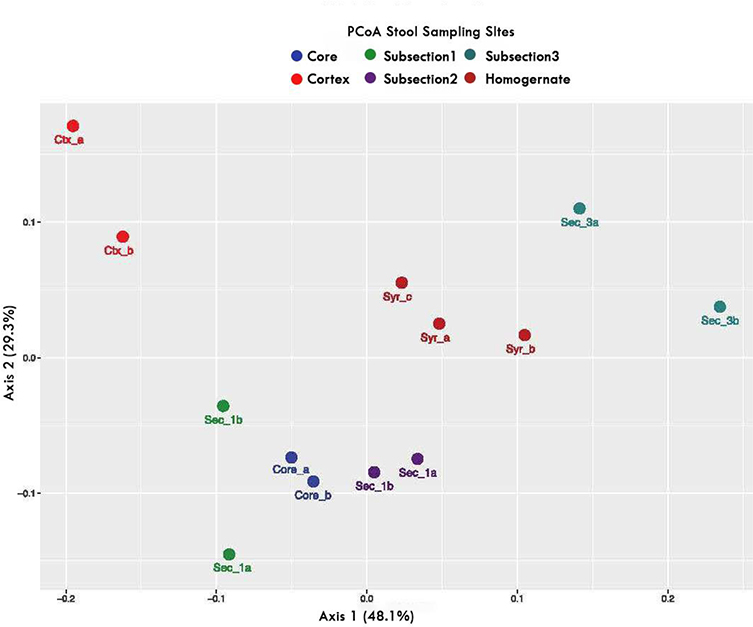
Principal coordinates analysis plot based on Bray-Curtis dissimilarity matrix for all stool sub-samples. The PCoA plot shows distinct clustering of the stool samples based on the region or segment of the stool from which they originated. The legend indicates the 6 different sampling sites, each one cm apart. The plot illustrates the coordinates for each individual sample. **Note:** 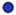 Core; 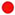 Cortex; 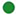 Subsection 1; 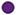 Subsection 2; 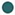 Subsection 3; 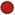 Homogernate.

To illustrate the magnitude of the heterogeneity within a single sample, the relative abundance of key taxa, representing low, medium and high relative abundances across the microbiomes analyzed, was compared in each section (Figure 3). Akkermansia was 3 times more abundant in the cortex compared to the other sections. Alistipes, *Bacteriodes* and Barnesiella had similar relative abundance profiles to each other in each section. *Collinsella* and Coprococcus have similar relative abundance profiles in each section. The relative abundance of *Bifidobacterium* was variable throughout the sections.

The average ratio of *Firmicutes*:*Bacteroidetes* was similar for Sections 1,2,3 syringe and core ranging from 1.45 to 2.5. However, the *Firmicutes*;*Bacteroidetes* ratio was significantly higher at 4.78 for the cortex making this subsample significantly different from the other sections (p=0.003) and low relative abundance represented in each section of the stool.

**Figure 4.**
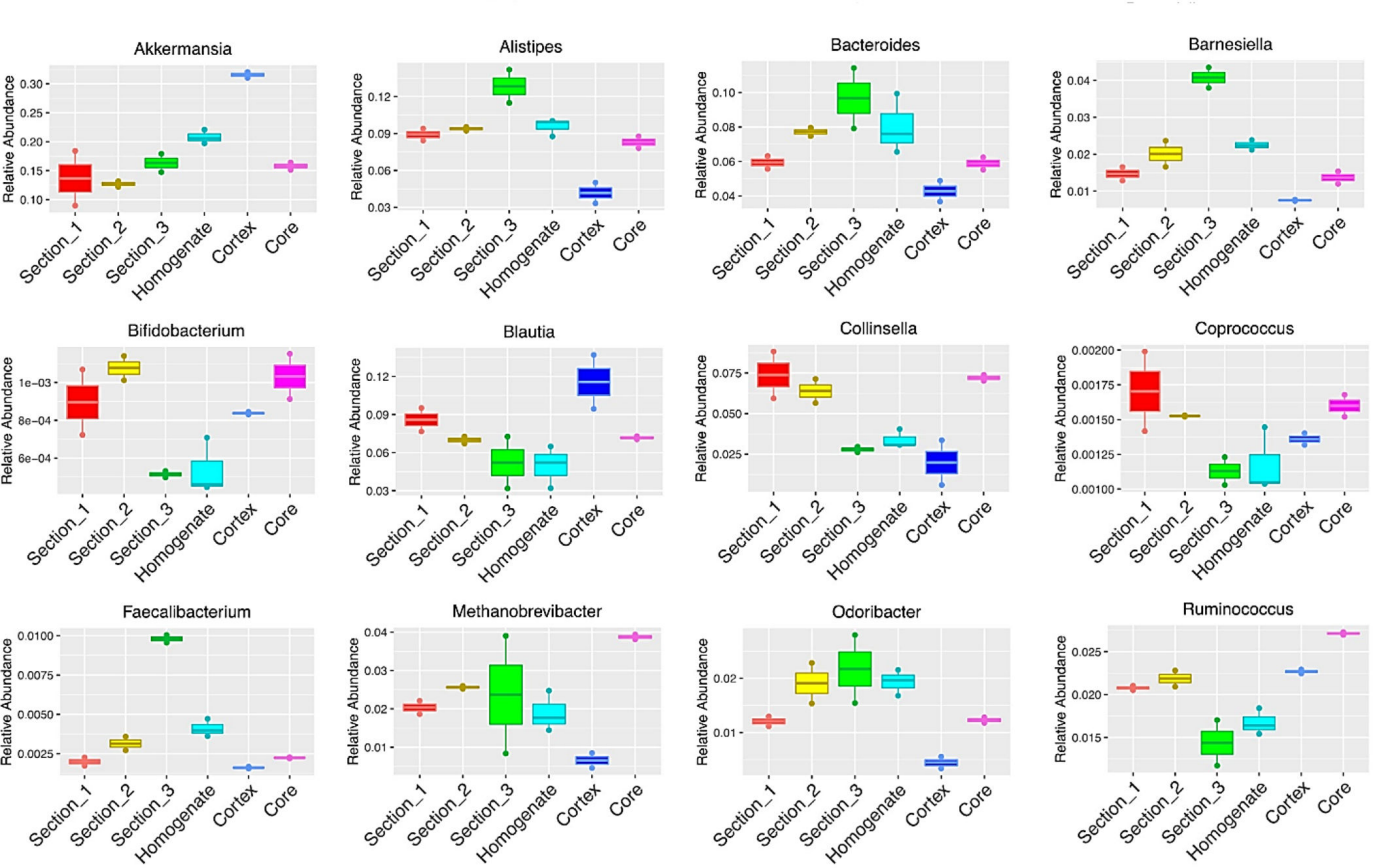
Differential abundance of taxa in dissected stool. The figure summarizes the distribution of 12 different genera present in high, medium. **Note:** 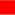 Section_1; 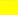 Section_2; 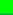 Section_3; 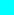 Homogenate; 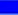 Cortex; 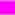 Core.

To determine how collection and processing effected microbiome composition, the second bowel movement was collected in the laboratory and processed as shown in Figure 4. The Relative Abundance (RA) for all phyla in 5 aliquots from each aliquot was compared. There were significant changes in the phyla RA for the fresh subsample while the RA of these five phyla remained stable in the 4C and frozen subsamples (Figure 4). At T10, *Firmicutes* and *Actinobacteria* were highly abundant. Within 2 minutes, the RA of *Actinobacteria* and *Firmicutes* began to decrease while the RA of *Bacteroidetes* and Proteobacteria increased. By 25 minutes, the RA of the *Bacteroidetes* increased from 0.014 at T10 to 0.37 at T25, representing a 95.85% increase. *Actinobacteria* decreased from 0.21 to 0.04, an 80% decrease in 15 minutes (from T10 to T25). *Firmicutes* decreased from 0.75 to 0.53 or 28.95%. The Proteobacteria increased RA from 0.0112 at T10 to 0.0368 at T25, a 69.6% increase. There was a significant loss in the Proteobacteria and an increased representation of *Actinobacteria* in all aliquots of the frozen subsample compared to the fresh and 4C subsamples (p<0.01). Proteobacteria RA differed significantly (p<0.05) between fresh and frozen subsamples. Proteobacteria RA in the frozen subsample averaged 0.0046 ± 0.0012, ranging from 0.0035 at T10 to 0.0045 at T25. This represents greater than 1 log reduction in RA when compared with the fresh subsample. There were slight variations in RA of the five major phyla in the 4C aliquots, but these differences were not significant.

**Figure 5.**
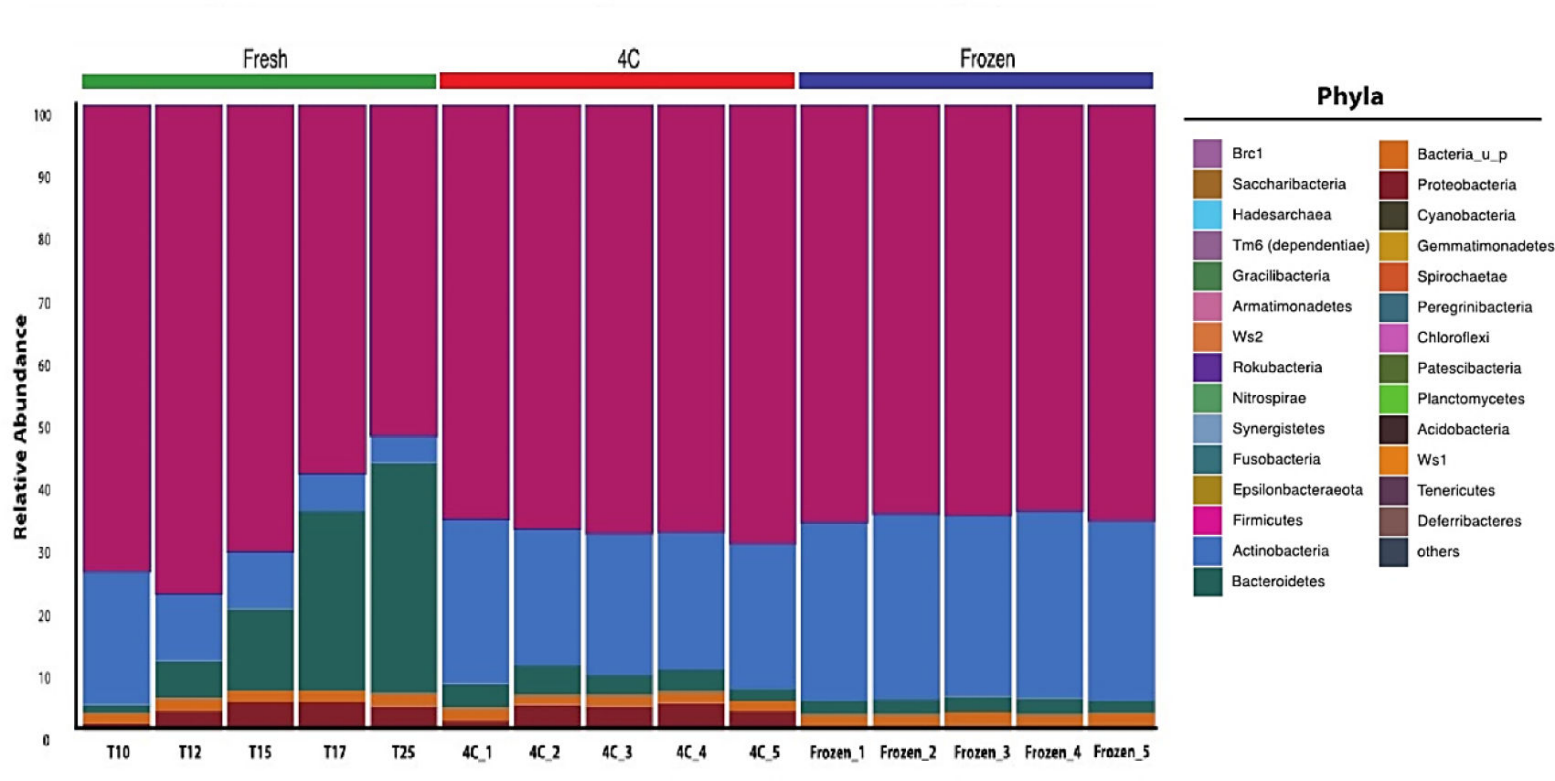
Taxonomic bar graph at the Phylum level for aliquots analyzed from three different sample processing methods. Relative abundance as a percent of total phyla was plotted as a stacked bar graph to illustrate the variations in relative abundance of individual phyla based on sample processing protocol and sampling time. **Note**: 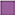 Brc 1; 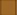 Saccharibacteria; 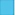 Hadesarchaea; 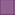 Tm6 (dependentiae); 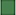 *Gracilibacteria*; 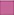 Armatimonadetes; 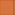 Ws2; 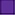 Rokubacteria; 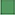 Nitrospirae; 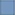 Synergistetes; 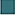 *Fusobacteria*; 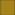 Epsilonbacteraeota; 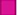 *Firmicutes*; 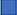 *Actinobacteria*; 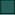 *Bacteroidetes*; 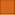 Bacteria_u_p; 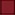 Proteobacteria; 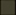 *Cyanobacteria*; 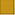 *Gemmatimonadetes*; 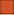 *Spirochaetae*; 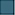 Peregrinibacteria; 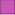 *Chloroflexi*; 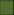 Patescibacteria; 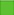 *Planctomycetes*; 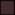 *Acidobacteria*; 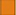 Ws1; 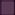 Tenericutes; 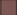 Deferribacteres; 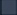 others.

To determine the effect of processing time on the *Firmicutes*:*Actinobacteria* ratio in the stool sample, 5 aliquots taken 10 minutes (T10), 12 minutes (T12), 15 minutes (T15), 17 minutes (T17) and 25 minutes (T25) were sequenced. At T10, *Firmicutes* and *Actinobacteria* were highly abundant (Figure 5). Within 2 minutes, the Relative Abundance (RA) of *Actinobacteria* and *Firmicutes* began to decrease while the RA of *Bacteroidetes* and Proteobacteria increased. At 25 minutes, the RA of the *Bacteroidetes* increased from 0.014 at T10 to 0.37 at T25, representing a 95.85% increase. Conversely, the *Firmicutes* decreased from 0.75 to 0.53 or 28.95%.

**Figure 6.**
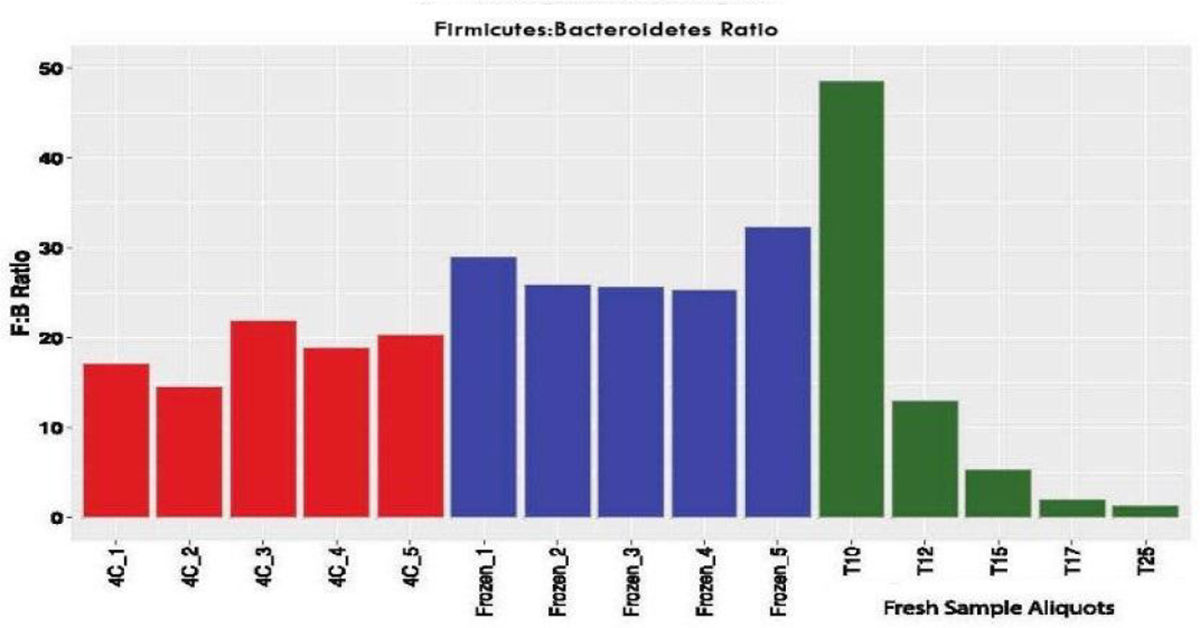
*Firmicutes* to *Bacteroidetes* ratio for aliquots analyzed from three different sample processing protocols. The ratio of *Firmicutes*:*Bacteroidetes* (F:B) was plotted as a side-by-side bar graph to illustrate the variations in F:B ratio for each sample processing protocol and sampling time.

Similarly, *Actinobacteria* decreased from 0.21 to 0.04, an 80% decrease in 15 minutes (from T10 to T25). The Proteobacteria showed a similar upward trend in RA to that of the *Bacteroidetes*. At T10, the calculated RA was 0.0112 and at T25 was 0.0368 or a 69.6% increase. The ratio of *Firmicutes*:*Bacteroidetes* changed from 48:6 at T10 to 1:43 at T25. These results are summarized in Figure 6.

Multidimensional scaling Analysis (MDA) using Bray-Curtis distance matrix from QIIME2 workflow was conducted on all replicate samples of the fresh, 4C and frozen workflows (Figure 6) ^[18,23,24]^. The results indicate that both the 4C and frozen aliquots group together as cohorts, reflecting the microbiome homogeneity of the aliquots within the cohort. Fresh sample T10, the closest sample to a fresh stool clustered with the 4C cohort. The remaining four aliquots (T12 – T25) progressively separate from the 4C cohort along Axis 2.3.2.

The Wilkinson rank sum test for each cohort was calculated and the results indicate that the alpha diversity of the microbiome, as measured using the Shannon And Simpson metrics, were significantly different from each other. The results are summarized in Table 1.

## DISCUSSION

It is apparent from the results illustrated in Figures 1 and 2 that the human stool, at least in this study, is heterogeneous throughout its length. Such heterogeneity has also been reported in similar, independent studies ^[32–34]^. As such, the microbiome composition can vary depending on the sampling site (e.g. outside surface or inner portions of the stool) as well the location of sampling along the length of the stool. This heterogeneity can be caused by diet, stress and many other environmental factors ^[29]^. Fecal output in healthy individuals average 1.20 defecations per 24 hours period, with variations from less than one bowel movement per day to more than 2 days per bowel movement, depending upon the transit time of the forming stool ^[29]^. This can significantly affect the composition of the microbiome during formation.

The sampling location of the stool can also have an impact on detection and identification of biomarkers. One salient example is that of bacteria such as *Akkermansia muciniphila*, a bacterium that predominantly thrive on the mucin layers of the intestinal epithelium, where continuous mucin production by the goblet cells and mucus desquamation occur and promote the growth of this bacterium ^[35]^. This bacterium has been recognized as a biomarker for inflammation and spegut health and suggested for use as a probiotic strain to promote gut health and immunity ^[36–39]^. In our study, it is apparent that the RA of *Akkermansia* in the fecal cortex (mean=0.54) is 3.75 times greater than in samples from the stool’s core (mean=0.16).

Similarly, in our studies, the ratio of *Firmicutes* to *Bacteroidetes* (F:B) varied greatly from where the sample was taken and ranged from 5.78 in the stool’s cortical sample and 1.12 in subsample 3 (Figure 2). These data are important to note as the F:B ratio has been used repeatedly as an indicator of gut health and dysbiosis ^[40,41]^.

These results indicate that the null hypothesis, that the stool is homogeneous, is false and as such, fully representative samples must be taken. Fecal samples collected by swabs or wipes would bias the relative abundance of bacteria, depending upon where the swab or wipe sample is taken. To ensure that a representative sample is taken, collection of whole stools with subsequent homogenization can normalize the microbiome composition and yield a representative sample from which the microbiome can be evaluated.

The results in Figure 4 indicate cooling the collected whole stool is essential to the stabilization and preservation of the microbiome composition. In this study, a single stool was collected at the laboratory, homogenized at room temperature and immediately split into three groups: Fresh, 4C and frozen. The fresh group was maintained at room temperature and random aliquots taken for analysis over a period of 25 minutes. The 4C sample was stabilized at 4°C then random aliquots were taken over a similar time period. Finally, the frozen samples were stored in dry ice for 24 hours, thawed at 4°C for 24 hours and finally 5 samples taken over a 25-minute period.

The sample exhibiting the most variability was the fresh sample. It can be inferred that this sample, which was at body temperature when collected, cooled slowly over the 25 minute sampling period at room temperature. During this time, significant changes in key biomarkers such as F:B ratio (Figure 5) along with changes in microbiome composition can be observed (Figure 6). The microbiome composition of the 4°C and dry ice stabilized samples exhibited no such dramatic changes in composition over a similar, 25 minute sampling period.

Oxygen exposure during sample preparation adversely impacts fecal bacterial communities, primarily the strict anaerobes ^[42]^. Approximately 50% of bacterial content of stool processed immediately under strict anaerobic conditions is non-viable ^[30]^. Homogenization in ambient air or freeze-thaw reduces viability to 19% and 23% respectively. Processing of samples in ambient air can result in several-fold reduction in the abundance of important commensal taxa, including the highly butyrogenic species *Faecalibacterium prausnitzii*, Subdoligranulum variable and Eubacterium hallii. The adverse impact of atmospheric oxygen exposure can reduce those species of bacteria involved Short Chain Fatty Acids (SCFA) biosynthesis. Conversely, while reducing alpha diversity, freeze-thaw does not significantly alter viable microbiota composition ^[42,30]^. These effects are more notable with stools processed at room temperature than at colder temperatures.

Even brief periods of storage of fecal samples at room temperature can impact the microbiome composition of samples ^[31]^. Gorzelak, et al. found significant differences in the major phyla of the gut, *Bacteroidetes* and *Firmicutes*, after 30 minutes compared to 15 minutes of storage at room temperature ^[31]^. Our own studies have shown that even 15 minutes at room temperature prior to refrigeration can affect the *Firmicutes*:*Bacteroidetes* (F:B) ratio (Figure 5). These changes can occur as a result of differential microbial growth, degradation of genomic DNA present in the stools and/or death of strict anaerobes.

It is essential that the stool be stabilized and the cooling period from fecal deposition to storage be minimized. This effect can be observed as changes in F:B ratios in Figure 6 in samples maintained at room temperature (T10–T25) and those cooled (4C) or placed in dry ice (frozen). It is apparent that the F:M ratio changes markedly from T10-T25 in the fresh samples. It is also notable that when freshly deposited fecal samples are stabilized by placing them immediately on freezer bricks or on dry ice, the F:B ratio remains stable throughout the fecal sample processing steps.

While it can be argued that microbiome analysis involves measuring microbiome DNA composition rather than community viability, DNA resulting from dead or damaged anaerobes can be degraded by aerotolerant and facultative anaerobes in the stool, thus, effectively reducing the relative abundance of the anaerobes in subsequent microbiome composition analysis.

It is apparent from the results, that the microbiome of freshly collected stool samples changes in composition rapidly if not stabilized by cooling or freezing as soon as practically possible. It is also apparent, that the method of microbiome stabilization (i.e., 4°C or dry ice) has an impact on microbiome composition. Wilkinson rank sum tests of alpha diversity metrics (Simpson and Shannon) show significant differences (p<0.05) between the two methods of stabilization (Table 1). When comparing the first two time points (T10 and T12) only of the Fresh sample collection, there is no significant difference in the Wilkinson rank sum test between the Fresh (T10 and T12) and the 4°C stool stabilization process (p=1.21). Conversely, when comparing the two methods of stool stabilization (4°C and dry ice), there is a significant difference between these two processes (p=0.0090). Similarly, PERMANOVA analyses of beta diversity of these two methods also show a significant difference (p=0.006).

While the stabilization of the stool by rapid cooling is essential for microbiome stabilization and preservation, freezing in dry ice, followed by a period of thawing will cause changes in microbiome composition, including the four major phyla represented in the human gut microbiome (*Actinobacteria*, *Bacteroidetes*, *Firmicutes* and Proteobacteria) ^[43–46]^. While immediate freezing has been long considered the best practice for sample preservation for microbiome studies, in our hands, freezing samples in dry ice result in a higher, significant increase (p<0.05) in *Actinobacteria* as compared to the 4°C sample as well as a higher F:B ratio.

## CONCLUSIONS

1. The human stool is not homogeneous in microbiome composition and as such, whole stools must be collected to capture the actual microbial composition of the human stool to ensure the integrity of the research, diagnostics and biomarker identification.
2. Collection of the entire bowel movement can be done from the comfort and privacy of the home.
3. Stabilization of the bowel movement at 4°C ensures the sample is as representative to the freshly evacuated bowel movement at possible.
4. Fecal subsamples (e.g. swabs) are not representative of the entire gut microbiome and samples that are not collected properly further bias the microbial profile. This is critical since research and biomarker discovery of the gut microbiome requires the availability of provenanced fecal sample that is representative of the gut microbiome and can be used in a variety of platforms. Convenient, at home collection of the entire bowel movement provides sufficient sample to homogenize and aliquot into multiple neat aliquots for testing in genomic, transcriptomic, metabolomic, lipidomic, culturomics, exposomics and phenomics. Although microbial sequencing surveys will continue to advance the field, microbiome research is beginning to focus on the function and mechanistic aspects of microbial communities. Therefore, collection of the entire bowel movement allows study of the microbiome on multiple platforms and concatenation of the all the microbiome data.

## Supplementary Material

References RRJMB| Volume 12 | Issue 1|March, 2023

Table RRJMB| Volume 12 | Issue 1|March, 2023

Supplementary file RRJMB| Volume 12 | Issue 1|March, 2023

Text RRJMB| Volume 12 | Issue 1|March, 2023

Figures RRJMB| Volume 12 | Issue 1|March, 2023

## Figures and Tables

**Figure 7.**
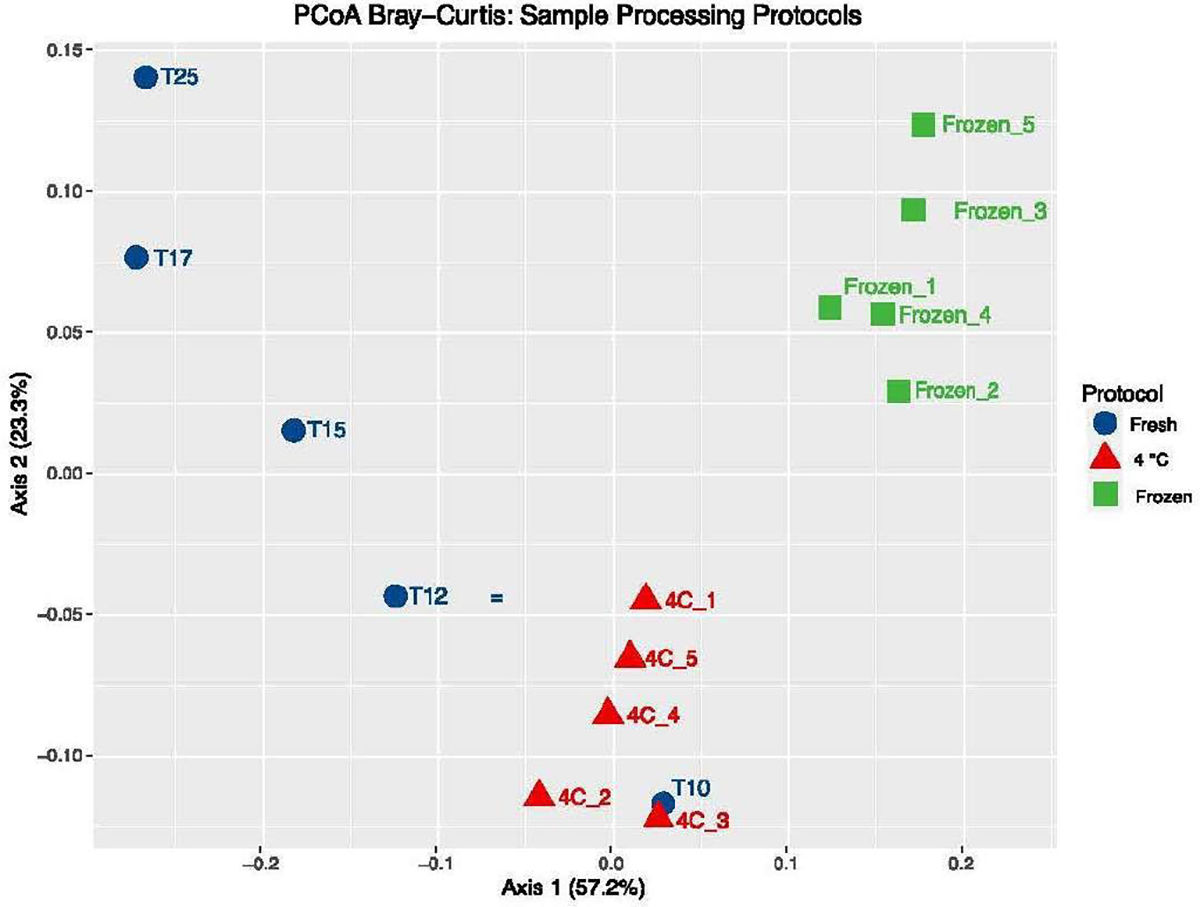
PCoA analysis of for aliquots analyzed from three different sample processing protocols. The PCoA plot shows distinct clustering of the stool samples based on the sample processing protocol used. The legend indicates the 3 different sampling processing protocols used in this study. The plot illustrates the coordinates for each individual sample. Note: 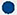 fresh; 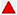 4°C 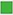 frozen.

**Table 1.** Wilkinson rank sum test of alpha diversity and PERMANOVA analysis of beta diversity for the various sample processing protocols.

Wilcoxon rank sum test (alpha diversity)				
	Shannon		Simpson	
Cohorts	Statistic	P-value*	Statistic	P-value*
**Fresh ↔ 4C**	−1.985	0.0472	−2.611	0.009
**Fresh ↔ Frozen**	1.3578	0.1745	2.6112	0.009
**4C ↔ Frozen**	2.6112	0.009	2.6112	0.009
PERMANOVA Analysis (beta diversity)				
Cohorts	Statistic	P-value*	-	-
**Fresh ↔ 4C**	1.97	0.043	-	-
**Fresh ↔ Frozen**	4.433	0.006	-	-
**4C ↔ Frozen**	2.567	0.005	-	-

## Data Availability

The datasets used and/or analyzed are available from the correspond ing author upon request.
