# Supplementary material for: Accelerating Gut Microbiome Research with Robust Sample Collection
Source: Res Rev J Microbiol Biotechnol. Author manuscript; Available in PMC 2023 Jun 29. (PMC10308701)
Supplement: References RRJMB| Volume 12 | Issue 1|March, 2023 [file NIHMS1904187-supplement-References_RRJMB__Volume_12___Issue_1_March__2023.docx]

**REFERENCES**

1. McDonald D, et al. [American gut: An open platform for citizen science microbiome research](https://journals.asm.org/doi/full/10.1128/mSystems.00031-18). Msystems. 2018;3:e00031-18. [[Crossref](https://doi.org/10.1128/mSystems.00031-18)][[Google Scholar](https://scholar.google.com/scholar?cluster=3942713517699842464&hl=en&as_sdt=0,5)][[Pubmed](https://pubmed.ncbi.nlm.nih.gov/29795809/)]
2. Barbara AM, et al. [A framework for human microbiome research](https://www.nature.com/articles/nature11209#ethics). Nature. 2012;486:215-221. [[Crossref](https://doi.org/10.1038/nature11209)][[Google Scholar](https://scholar.google.com/scholar?cluster=1796324860816792435&hl=en&as_sdt=0,5)][[Pubmed](https://pubmed.ncbi.nlm.nih.gov/22699610/)]
3. Sinha R, et al. [Collecting fecal samples for microbiome analyses in epidemiology studies](https://aacrjournals.org/cebp/article/25/2/407/115739/Collecting-Fecal-Samples-for-Microbiome-Analyses). Cancer Epidemiol Biomark Preven. 2016;25:407-416. [[Crossref](https://doi.org/10.1158/1055-9965.EPI-15-0951)][[Google Scholar](https://scholar.google.com/scholar?cluster=14625833897026957525&hl=en&as_sdt=0,5)][[Pubmed](https://pubmed.ncbi.nlm.nih.gov/26604270/)]
4. Reyman M, et al. [Rectal swabs are a reliable proxy for faecal samples in infant gut microbiota research based on 16S-rRNA sequencing](https://www.nature.com/articles/s41598-019-52549-z). Sci Rep. 2019;9:1-8. [[Crossref](https://doi.org/10.1038/s41598-019-52549-z)][[Google Scholar](https://scholar.google.com/scholar?cluster=10007801451673047582&hl=en&as_sdt=0,5)][[Pubmed](https://pubmed.ncbi.nlm.nih.gov/31690798/)]
5. Biehl LM, et al. [Usability of rectal swabs for microbiome sampling in a cohort study of hematological and oncological patients](https://journals.plos.org/plosone/article?id=10.1371/journal.pone.0215428). PloS one. 2019;14:e0215428. [[Crossref](https://doi.org/10.1371/journal.pone.0215428)][[Google Scholar](https://scholar.google.com/scholar?cluster=18409261922344429678&hl=en&as_sdt=0,5)][[Pubmed](https://pubmed.ncbi.nlm.nih.gov/30986251/)]
6. Kumar R, et al. [Getting started with microbiome analysis: Sample acquisition to bioinformatics](https://currentprotocols.onlinelibrary.wiley.com/doi/abs/10.1002/0471142905.hg1808s82). Curr protoc Human Genet. 2014;82:18.8.1-18.8.29. [[Crossref](https://doi.org/10.1002/0471142905.hg1808s82)][[Google Scholar](https://scholar.google.com/scholar?cluster=11068009766542028580&hl=en&as_sdt=0,5)][[Pubmed](https://pubmed.ncbi.nlm.nih.gov/25042718/)]
7. Dubois G, et al. [The Inuit gut microbiome is dynamic over time and shaped by traditional foods](https://microbiomejournal.biomedcentral.com/articles/10.1186/s40168-017-0370-7). Microbiome. 2017;5:1-12. [[Crossref](https://doi.org/10.1186/s40168-017-0370-7)][[Google Scholar](https://scholar.google.com/scholar?cluster=17096592300680304994&hl=en&as_sdt=0,5)][[Pubmed](https://www.ncbi.nlm.nih.gov/pmc/articles/PMC5689144/)]
8. Kurian SM, et al. [Feasibility and comparison study of fecal sample collection methods in healthy volunteers and solid organ transplant recipients using 16S rRNA and metagenomics approaches](https://www.liebertpub.com/doi/abs/10.1089/bio.2020.0032). Biopreserv Biobank. 2020;18:425-440. [[Crossref](https://doi.org/10.1089/bio.2020.0032)][[Google Scholar](https://scholar.google.com/scholar?cluster=1384782359369940717&hl=en&as_sdt=0,5)][[Pubmed](https://pubmed.ncbi.nlm.nih.gov/32833508/)]
9. Wu WK, et al. [Optimization of fecal sample processing for microbiome study-the journey from bathroom to bench](https://www.sciencedirect.com/science/article/pii/S0929664617308574). J Formos Med Assoc. 2019;118:545-555. [[Crossref](https://doi.org/10.1016/j.jfma.2018.02.005)][[Google Scholar](https://scholar.google.com/scholar?cluster=13479845415701069889&hl=en&as_sdt=0,5)][[Pubmed](https://pubmed.ncbi.nlm.nih.gov/29490879/)]
10. Zackular JP, et al. [The human gut microbiome as a screening tool for colorectal cancer](https://aacrjournals.org/cancerpreventionresearch/article/7/11/1112/50234/The-Human-Gut-Microbiome-as-a-Screening-Tool-for). Cancer Prev Res. 2014;7:1112-1121. [[Crossref](https://doi.org/10.1158/1940-6207.CAPR-14-0129)][[Google Scholar](https://scholar.google.com/scholar?cluster=1844144084290281889&hl=en&as_sdt=0,5)][[Pubmed](https://pubmed.ncbi.nlm.nih.gov/25104642/)]
11. Costea PI, et al. [Towards standards for human fecal sample processing in metagenomic studies](https://www.nature.com/articles/nbt.3960). Nat Biotechnol. 2017;35:1069-1076. [[Crossref](https://doi.org/10.1038/nbt.3960)][[Google Scholar](https://scholar.google.com/scholar?cluster=15024281358139605931&hl=en&as_sdt=0,5)][[Pubmed](https://pubmed.ncbi.nlm.nih.gov/28967887/)]
12. Vogtmann E, et al. [Comparison of collection methods for fecal samples in microbiome studies](https://academic.oup.com/aje/article/185/2/115/2706021). Am J Epidemiol. 2017;185:115-123. [[Crossref](https://doi.org/10.1093/aje/kww177)][[Google Scholar](https://scholar.google.com/scholar?cluster=6712404822037423195&hl=en&as_sdt=0,5)][[Pubmed](https://pubmed.ncbi.nlm.nih.gov/27986704/)]
13. Watson EJ, et al. [Human faecal collection methods demonstrate a bias in microbiome composition by cell wall structure](https://www.nature.com/articles/s41598-019-53183-5). Sci Rep. 2019;9:1-8. [[Crossref](https://doi.org/10.1038/s41598-019-53183-5)][[Google Scholar](https://scholar.google.com/scholar?cluster=4428755710530672937&hl=en&as_sdt=0,5)][[Pubmed](https://pubmed.ncbi.nlm.nih.gov/31727963/)]
14. Wu WK, et al. [Optimization of fecal sample processing for microbiome study-the journey from bathroom to bench](https://europepmc.org/article/med/29490879). J Formos Med Assoc. 2018;118:545-555. [[Crossref](https://doi.org/10.1016/j.jfma.2018.02.005)][[Google Scholar](https://scholar.google.com/scholar?cluster=13479845415701069889&hl=en&as_sdt=0,5)][[Pubmed](https://pubmed.ncbi.nlm.nih.gov/29490879/)]
15. Sutherland VL, et al. [The gut microbiome and xenobiotics: Identifying knowledge gaps](https://academic.oup.com/toxsci/article/176/1/1/5862611). Toxicol Sci. 2020;176:1-10. [[Crossref](https://doi.org/10.1093/toxsci/kfaa060)][[Google Scholar](https://scholar.google.com/scholar?cluster=14437231829461651851&hl=en&as_sdt=0,5)][[Pubmed](https://www.ncbi.nlm.nih.gov/pmc/articles/PMC7850111/)]
16. Lewis SJ, et al. [Stool form scale as a useful guide to intestinal transit time](https://www.tandfonline.com/doi/abs/10.3109/00365529709011203). Scand J Gastroenterol. 1997;32:920-924. [[Crossref](https://doi.org/10.3109/00365529709011203)][[Google Scholar](https://scholar.google.com/scholar?cluster=15024292234756389326&hl=en&as_sdt=0,5)][[Pubmed](https://pubmed.ncbi.nlm.nih.gov/9299672/)]
17. Callahan BJ, et al. [DADA2: High-resolution sample inference from illumina amplicon data](https://www.nature.com/articles/nmeth.3869). Nat Methods. 2016;13:581-583. [[Crossref](https://doi.org/10.1038/nmeth.3869)][[Google Scholar](https://scholar.google.com/scholar?cluster=1592655680316646386&hl=en&as_sdt=0,5)][[Pubmed](https://www.ncbi.nlm.nih.gov/pmc/articles/PMC4927377/)]
18. Bolyen E, et al. [Reproducible, interactive, scalable and extensible microbiome data science using QIIME 2](https://www.nature.com/articles/s41587-019%200209-9). Nat Biotechnol. 2019;37:852-857. [[Crossref](https://doi.org/10.1038/s41587-019-0209-9)][[Google Scholar](https://scholar.google.com/scholar?cluster=6935821321202015575&hl=en&as_sdt=0,5)][[Pubmed](https://www.ncbi.nlm.nih.gov/pmc/articles/PMC7015180/)]
19. Quast C, et al. [The SILVA ribosomal RNA gene database project: Improved data processing and web-based tools](https://academic.oup.com/nar/article/41/D1/D590/1069277). Nucleic Acids Res. 2012;41:D590-D596. [[Crossref](https://doi.org/10.1093/nar/gks1219)][[Google Scholar](https://scholar.google.com/scholar?cluster=6892915615612580344&hl=en&as_sdt=0,5)][[Pubmed](https://pubmed.ncbi.nlm.nih.gov/23193283/)]
20. Shannon CE. [A mathematical theory of communication](https://dl.acm.org/doi/abs/10.1145/584091.584093). ACM Mob Comp Comm.2001;5:3-55. [[Crossref](https://doi.org/10.1145/584091.584093)][[Google Scholar](https://scholar.google.com/scholar?cluster=517179926353339293&hl=en&as_sdt=0,5)]
21. Chao A. [Nonparametric estimation of the number of classes in a population](http://chao.stat.nthu.edu.tw/wordpress/paper/1984_ScandJStatist_11_P265.pdf). Scand J Statist. 1984;11:265-270. [[Google Scholar](https://scholar.google.com/scholar?cluster=5883280989260428684&hl=en&as_sdt=0,5)]
22. Pielou EC. [The measurement of diversity in different types of biological collections](https://www.sciencedirect.com/science/article/abs/pii/0022519366900130). J Theor Biol. 1966;13:131-144. [[Crossref](https://doi.org/10.1016/0022-5193(66)90013-0)][[Google Scholar](https://scholar.google.com/scholar?cluster=17625591749210567681&hl=en&as_sdt=0,5)]
23. Cox MAA, et al. [Multidimensional scaling](https://link.springer.com/chapter/10.1007/978-3-540-33037-0_14). Handbook of data visualization. 2008:315-347. [[Google Scholar](https://scholar.google.com/scholar?hl=en&as_sdt=0%2C5&q=Multidimensional+scaling%2C+in+Handbook+of+data+visualization&btnG=)]
24. Bray JR, et al. [An ordination of the upland forest communities of southern wisconsin](https://www.jstor.org/stable/1942268). Ecol Monogr. 1957;27:325-349. [[Crossref](https://doi.org/10.2307/1942268)][[Google Scholar](https://scholar.google.com/scholar?cluster=8621521593477201483&hl=en&as_sdt=0,5)]
25. Jaccard P. [Comparative study of the floral distribution in a portion of the alps and the jura](https://cir.nii.ac.jp/crid/1570009750546179712). Bull Soc Vaudoise Sci Nat. 1901;37:547-579. [[Google Scholar](https://scholar.google.com/scholar?cluster=6523824902603703002&hl=en&as_sdt=0,5)]
26. Mann HB, et al. [On a test of whether one of two random variables is stochastically larger than the other](https://www.jstor.org/stable/2236101). Ann Math Stat. 1947;18:50-60. [[Google Scholar](https://scholar.google.com/scholar?cluster=17213226055159238535&hl=en&as_sdt=0,5)]
27. Anderson MJ. [A new method for non-parametric multivariate analysis of variance](https://onlinelibrary.wiley.com/doi/abs/10.1111/j.1442-9993.2001.01070.pp.x). Aus Ecol. 2001;26:32-46. [[Crossref](https://doi.org/10.1111/j.1442-9993.2001.01070.pp.x)][[Google Scholar](https://scholar.google.com/scholar?cluster=756643875316980061&hl=en&as_sdt=0,5)]
28. Daniel WW. [The kruskal-wallis one-way analysis of variance by ranks](https://psych.unl.edu/psycrs/handcomp/hckw.PDF). Appl Nonpar Statis. 1990:226-234. [[Google Scholar](https://scholar.google.com/scholar?hl=en&as_sdt=0%2C5&q=Kruskal%E2%80%93Wallis+one-way+analysis+of+variance+by+ranks&btnG=)]
29. Rose C, et al. [The characterization of feces and urine: A review of the literature to inform advanced treatment technology](https://www.tandfonline.com/doi/full/10.1080/10643389.2014.1000761). Crit Rev Environ Sci Technol. 2015;45:1827-1879. [[Crossref](https://doi.org/10.1080/10643389.2014.1000761)][[Google Scholar](https://scholar.google.com/scholar?cluster=14676683818740800270&hl=en&as_sdt=0,5)][[Pubmed](https://www.ncbi.nlm.nih.gov/pmc/articles/PMC4500995/)]
30. Papanicolas LE, et al. [Bacterial viability in faecal transplants: Which bacteria survive?](https://www.sciencedirect.com/science/article/pii/S2352396419300957). Ebiomed. 2019;41:509-516. [[Crossref](https://doi.org/10.1016/j.ebiom.2019.02.023)][[Google Scholar](https://scholar.google.com/scholar?cluster=4539710941427079040&hl=en&as_sdt=0,5)][[Pubmed](https://pubmed.ncbi.nlm.nih.gov/30796005/)]
31. Gorzelak MA, et al. [Methods for improving human gut microbiome data by reducing variability through sample processing and storage of stool](https://journals.plos.org/plosone/article?id=10.1371/journal.pone.0134802). PLoS One. 2015;10:e0134802. [[Crossref](https://doi.org/10.1371/journal.pone.0134802)][[Google Scholar](https://scholar.google.com/scholar?cluster=13209001654736736283&hl=en&as_sdt=0,5)][[Pubmed](https://pubmed.ncbi.nlm.nih.gov/26252519/)]
32. Wu GD, et al. [Sampling and pyrosequencing methods for characterizing bacterial communities in the human gut using 16S sequence tags](https://bmcmicrobiol.biomedcentral.com/articles/10.1186/1471-2180-10-206). BMC Microbiol. 2010;10:206. [[Crossref](https://doi.org/10.1186/1471-2180-10-206)][[Google Scholar](https://scholar.google.com/scholar?cluster=8518936164574268288&hl=en&as_sdt=0,5)][[Pubmed](https://pubmed.ncbi.nlm.nih.gov/20673359/)]
33. Huson DH, et al. [A simple statistical test of taxonomic or functional homogeneity using replicated microbiome sequencing samples](https://www.sciencedirect.com/science/article/pii/S0168165616315760). J Biotechnol. 2017;250:45-50. [[Crossref](https://doi.org/10.1016/j.jbiotec.2016.10.020)][[Google Scholar](https://scholar.google.com/scholar?cluster=11066055631202277596&hl=en&as_sdt=0,5)][[Pubmed](https://pubmed.ncbi.nlm.nih.gov/27984120/)]
34. Gratton J, et al. [Optimized sample handling strategy for metabolic profiling of human feces](https://pubs.acs.org/doi/abs/10.1021/acs.analchem.5b04159). Anal Chem. 2016;88:4661-4668. [[Crossref](https://doi.org/10.1021/acs.analchem.5b04159)][[Google Scholar](https://scholar.google.com/scholar?cluster=16940276721305771233&hl=en&as_sdt=0,5)][[Pubmed](https://pubmed.ncbi.nlm.nih.gov/27065191/)]
35. Herreweghen FV, et al. [Mucin degradation niche as a driver of microbiome composition and *Akkermansia muciniphila* abundance in a dynamic gut model is donor independent](https://academic.oup.com/femsec/article/94/12/fiy186/5101425). FEMS Microbiol Ecol. 2018;94: fiy186. [[Crossref](https://doi.org/10.1093/femsec/fiy186)][[Google Scholar](https://scholar.google.com/scholar?cluster=2576928959520003445&hl=en&as_sdt=0,5)][[Pubmed](https://pubmed.ncbi.nlm.nih.gov/30239657/)]
36. Ouyang J, et al. [The Bacterium *Akkermansia muciniphila*: A sentinel for gut permeability and its relevance to HIV-related inflammation](https://www.frontiersin.org/articles/10.3389/fimmu.2020.00645/full). Front Immunol. 2020;11:645. [[Crossref](https://doi.org/10.3389/fimmu.2020.00645)][[Google Scholar](https://scholar.google.com/scholar?cluster=5881827216361740762&hl=en&as_sdt=0,5)][[Pubmed](https://pubmed.ncbi.nlm.nih.gov/32328074/)]
37. Jayachandran M, et al. [A critical review of the relationship between dietary components, the gut microbe *Akkermansia muciniphila*, and human health](https://www.tandfonline.com/doi/abs/10.1080/10408398.2019.1632789). Crit Rev Food Sci Nutr. 2020;60:2265-2276. [[Crossref](https://doi.org/10.1080/10408398.2019.1632789)][[Google Scholar](https://scholar.google.com/scholar?cluster=13641632323867568320&hl=en&as_sdt=0,5)][[Pubmed](https://pubmed.ncbi.nlm.nih.gov/31257904/)]
38. Zhang T, et al. [*Akkermansia muciniphila* is a promising probiotic](https://ami-journals.onlinelibrary.wiley.com/doi/full/10.1111/1751-7915.13410). Microb Biotechnol. 2019;12:1109-1125. [[Crossref](https://doi.org/10.1111/1751-7915.13410)][[Google Scholar](https://scholar.google.com/scholar?cluster=14628584590226869962&hl=en&as_sdt=0,5)][[Pubmed](https://pubmed.ncbi.nlm.nih.gov/31006995/)]
39. Ansaldo E, et al. [*Akkermansia muciniphila* induces intestinal adaptive immune responses during homeostasis](https://www.science.org/doi/abs/10.1126/science.aaw7479). Science. 2019;364:1179-1184. [[Crossref](https://doi.org/10.1126/science.aaw7479)][[Google Scholar](https://scholar.google.com/scholar?cluster=17321086419865246579&hl=en&as_sdt=0,5)][[Pubmed](https://pubmed.ncbi.nlm.nih.gov/31221858/)]
40. Magne F, et al. [The *Firmicutes*/*Bacteroidetes* ratio: A relevant marker of gut dysbiosis in obese patients?](https://www.mdpi.com/2072-6643/12/5/1474). Nutrients. 2020;12:1474. [[Crossref](https://doi.org/10.3390/nu12051474)][[Google Scholar](https://scholar.google.com/scholar?cluster=6674437064303994918&hl=en&as_sdt=0,5)][[Pubmed](https://pubmed.ncbi.nlm.nih.gov/32438689/)]
41. Jasirwan COM, et al. [Correlation of gut Firmicutes/Bacteroidetes ratio with fibrosis and steatosis in patients with non-alcoholic fatty liver disease](https://www.jstage.jst.go.jp/article/bmfh/40/1/40_2020-046/_article). 2021;40:50-58. [[Crossref](https://doi.org/10.12938/bmfh.2020-046)][[Google Scholar](https://scholar.google.com/scholar?cluster=8108961723629043505&hl=en&as_sdt=0,5)][[Pubmed](https://pubmed.ncbi.nlm.nih.gov/33520569/)]
42. Chu ND, et al. [Profiling living bacteria informs preparation of fecal microbiota transplantations](https://journals.plos.org/plosone/article?id=10.1371/journal.pone.0170922). PloS one. 2017;12:e0170922. [[Crossref](https://doi.org/10.1371/journal.pone.0170922)][[Google Scholar](https://scholar.google.com/scholar?cluster=2770148747573161289&hl=en&as_sdt=0,5)][[Pubmed](https://pubmed.ncbi.nlm.nih.gov/28125667/)]
43. Molbak L, et al. [Freezing at -80ºC distorts the DNA composition of bacterial communities in intestinal samples](https://www.caister.com/backlist/ciim/v/v7/05.pdf). Curr Issues in Intestinal Microbiol. 2006;7:29-34. [[Google Scholar](https://scholar.google.com/scholar?cluster=142618937630498944&hl=en&as_sdt=0,5)][[Pubmed](https://pubmed.ncbi.nlm.nih.gov/16570697/)]
44. Walker VK, et al. [Freeze-thaw tolerance and clues to the winter survival of a soil community](https://journals.asm.org/doi/full/10.1128/AEM.72.3.1784-1792.2006). Appl Environ Microbiol. 2006;72:1784-1792. [[Crossref](https://doi.org/10.1128/AEM.72.3.1784-1792.2006)][[Google Scholar](https://scholar.google.com/scholar?cluster=2486171333969174712&hl=en&as_sdt=0,5)][[Pubmed](https://pubmed.ncbi.nlm.nih.gov/16517623/)]
45. Choo JM, et al. [Sample storage conditions significantly influence faecal microbiome profiles](https://www.nature.com/articles/srep16350). Sci Rep. 2015;5:16350. [[Crossref](https://doi.org/10.1038/srep16350)][[Google Scholar](https://scholar.google.com/scholar?cluster=1613854532715587347&hl=en&as_sdt=0,5)][[Pubmed](https://pubmed.ncbi.nlm.nih.gov/26572876/)]
46. Ezzy AC, et al. [Storage and handling of human faecal samples affect the gut microbiome composition: A feasibility study](https://www.sciencedirect.com/science/article/abs/pii/S0167701218308789). J Microbiol Methods. 2019;164:105668. [[Crossref](https://doi.org/10.1016/j.mimet.2019.105668)][[Google Scholar](https://scholar.google.com/scholar?cluster=17723312909307498819&hl=en&as_sdt=0,5)][[Pubmed](https://pubmed.ncbi.nlm.nih.gov/31302202/)]
