# Supplementary material for: Accelerating Gut Microbiome Research with Robust Sample Collection
Source: Res Rev J Microbiol Biotechnol. Author manuscript; Available in PMC 2023 Jun 29. (PMC10308701)
Supplement: Table RRJMB| Volume 12 | Issue 1|March, 2023 [file NIHMS1904187-supplement-Table_RRJMB__Volume_12___Issue_1_March__2023.docx]

**Table 1**. Wilkinson rank sum test of alpha diversity and PERMANOVA analysis of beta diversity for the various sample processing protocols.

| **Wilcoxon rank sum test (alpha diversity)** | | | | |
| --- | --- | --- | --- | --- |
|  | **Shannon** | | **Simpson** | |
| **Cohorts** | **Statistic** | **P-value^*^** | **Statistic** | **P-value^*^** |
| **Fresh ↔ 4C** | -1.985 | 0.0472 | -2.611 | 0.009 |
| **Fresh ↔ Frozen** | 1.3578 | 0.1745 | 2.6112 | 0.009 |
| **4C ↔ Frozen** | 2.6112 | 0.009 | 2.6112 | 0.009 |
| **PERMANOVA Analysis (beta diversity)** | | | | |
| **Cohorts** | **Statistic** | **P-value^*^** | - | - |
| **Fresh ↔ 4C** | 1.97 | 0.043 | - | - |
| **Fresh ↔ Frozen** | 4.433 | 0.006 | - | - |
| **4C ↔ Frozen** | 2.567 | 0.005 | - | - |
