# Supplementary material for: Accelerating Gut Microbiome Research with Robust Sample Collection
Source: Res Rev J Microbiol Biotechnol. Author manuscript; Available in PMC 2023 Jun 29. (PMC10308701)
Supplement: Supplementary file RRJMB| Volume 12 | Issue 1|March, 2023 [file NIHMS1904187-supplement-Supplementary_file_RRJMB__Volume_12___Issue_1_March__2023.docx]

**Supplementary Figure 1**. The “blind” reference sample passed QC based on taxonomic profile.


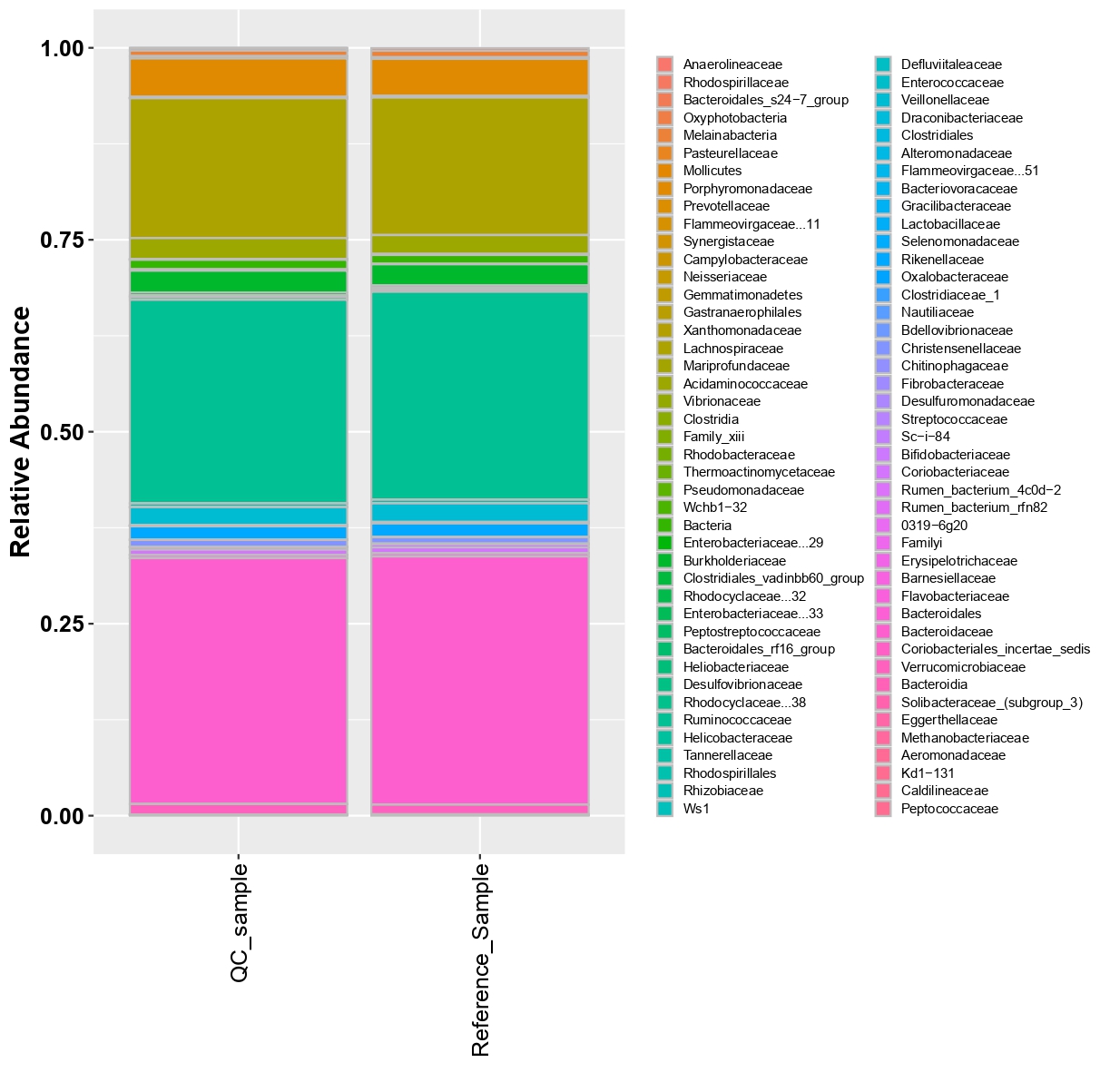


**Supplementary Table 1**. The average number of input-reads for DADA2 processing which passes denoising and chimera analysis.

| **Sample-ID** | **Input** | **Filtered** | **Percentage of input passed filter** | **Denoised** | **Non-Chimeric** | **Percentage of input non-chimeric** |
| --- | --- | --- | --- | --- | --- | --- |
| 06172020-QC | 82,474 | 78,152 | 94.76 | 76,408 | 53,291 | 64.62 |
| 06172020-TMX | 74,991 | 71,047 | 94.74 | 69,748 | 44,904 | 59.88 |
| 136310 | 86,057 | 80,965 | 94.08 | 79,978 | 34,588 | 40.19 |
| 136312 | 65,643 | 62,042 | 94.51 | 61,048 | 32,304 | 49.21 |
| 136316 | 76,569 | 72,162 | 94.24 | 70,872 | 40,890 | 53.40 |
| 136320 | 76,276 | 72,193 | 94.65 | 71,047 | 39,698 | 52.05 |
| 136330 | 72,171 | 68,245 | 94.56 | 67,175 | 35,866 | 49.70 |
| 136617 | 80,376 | 75,599 | 94.06 | 74,414 | 38,486 | 47.88 |
| 136620 | 62,738 | 59,255 | 94.45 | 58,182 | 33,528 | 53.44 |
| 136622 | 71,141 | 66,771 | 93.86 | 65,524 | 32,324 | 45.44 |
| 136625 | 78,531 | 73,942 | 94.16 | 72,937 | 40,663 | 51.78 |
| 136628 | 73,543 | 69,084 | 93.94 | 68,258 | 34,000 | 46.23 |
| 136738 | 79,258 | 74,529 | 94.03 | 73,396 | 34,267 | 43.23 |
| 136742 | 69,888 | 65,806 | 94.16 | 64,717 | 31,320 | 44.81 |
| 136745 | 80,182 | 75,224 | 93.82 | 74,309 | 34,311 | 42.79 |
| 136752 | 77,262 | 72,697 | 94.09 | 71,481 | 37,578 | 48.64 |
| 136756 | 70,144 | 65,903 | 93.95 | 64,952 | 33,963 | 48.42 |
| **Average** | **75,132** | **70,801** | **94.24** | **69,673** | **37,175** | **49.51** |
| **Standard Dev** | **6,052** | **5,678** | **31%** | **5,620** | **5,556** | **6.14** |
