# Supplementary material for: Accelerating Gut Microbiome Research with Robust Sample Collection
Source: Res Rev J Microbiol Biotechnol. Author manuscript; Available in PMC 2023 Jun 29. (PMC10308701)

**Figure 1**. Sample Processing Workflow. This figure shows the steps taken in sample collection and processing for comparing sample preparation protocols.


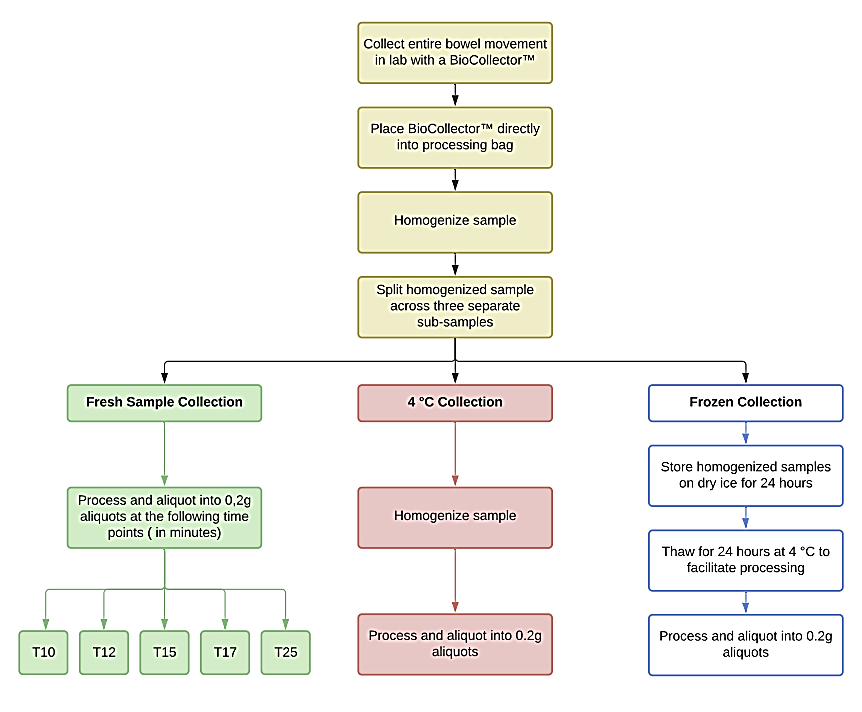


**Figure 2**. Schematic representation of the stool dissection study to evaluate the level of microbiome homogeneity of each section. Each section was processed independently and analysed for microbiome composition.


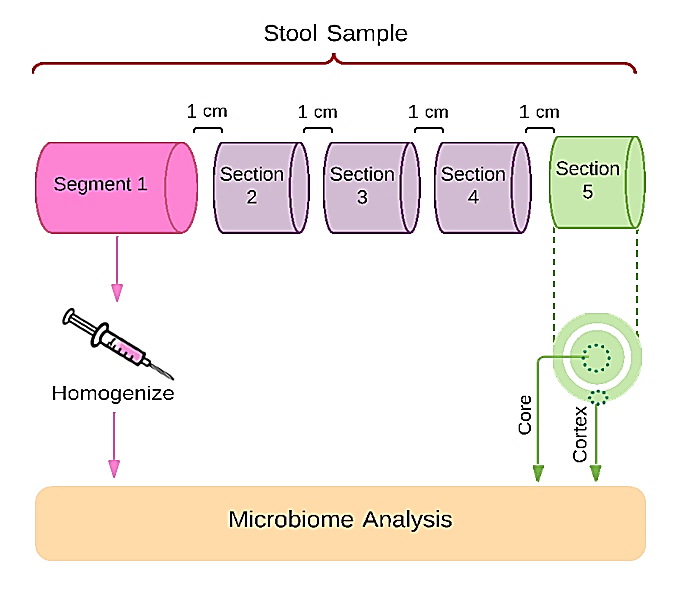


**Figure 3**. Principal coordinates analysis plot based on Bray-Curtis dissimilarity matrix for all stool sub-samples. The PCoA plot shows distinct clustering of the stool samples based on the region or segment of the stool from which they originated. The legend indicates the 6 different sampling sites, each one cm apart. The plot illustrates the coordinates for each individual sample. **Note**:
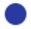
Core;
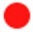
 Cortex;
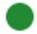
 Subsection 1;
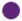
 Subsection 2;
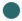
 Subsection 3;
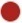
 Homogernate.


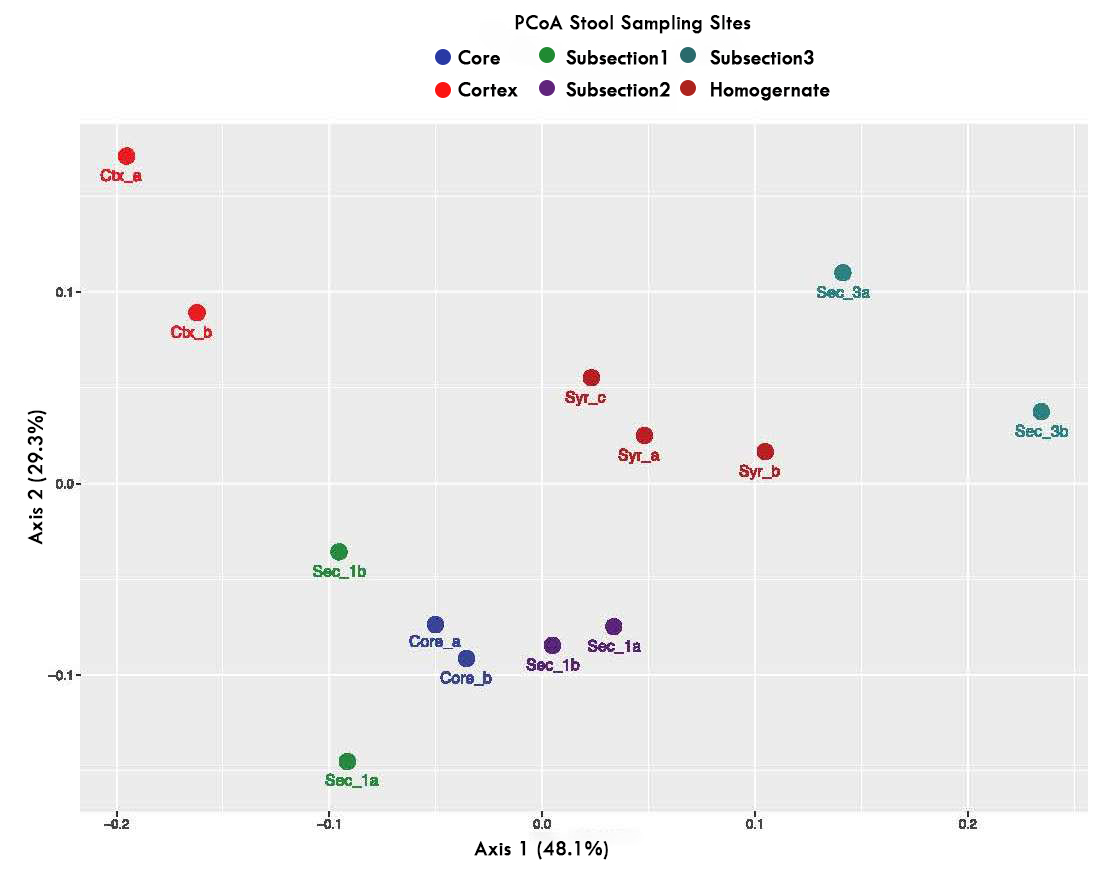


**Figure 4**. Differential abundance of taxa in dissected stool. The figure summarizes the distribution of 12 different genera present in high, medium. **Note**:
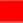
 Section_1;
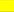
 Section_2;
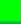
 Section_3;
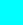
 Homogenate;
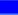
 Cortex;
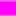
 Core.


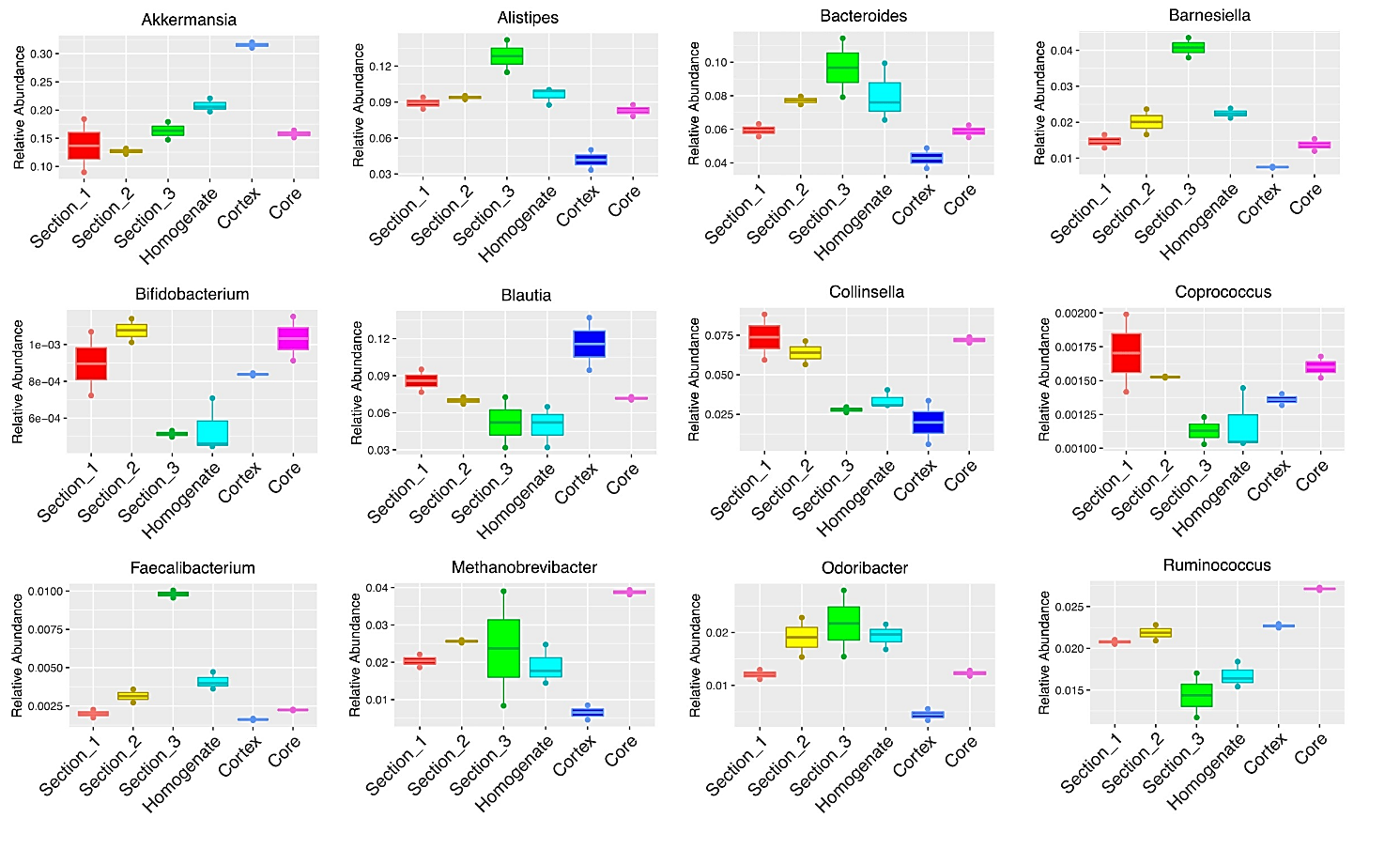


**Figure 5**. Taxonomic bar graph at the Phylum level for aliquots analyzed from three different sample processing methods. Relative abundance as a percent of total phyla was plotted as a stacked bar graph to illustrate the variations in relative abundance of individual phyla based on sample processing protocol and sampling time. **Note**:
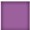
 Brc 1;
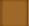
 Saccharibacteria;
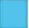
 Hadesarchaea;
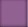
 Tm6 (dependentiae);
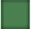
 *Gracilibacteria*;
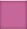
 Armatimonadetes;
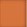
 Ws2;
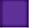
 Rokubacteria;
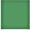
 Nitrospirae;
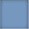
 Synergistetes;
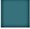
 *Fusobacteria*;
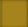
 Epsilonbacteraeota;
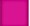
 *Firmicutes*;
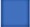
 *Actinobacteria*;
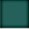
 *Bacteroidetes*;
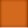
 Bacteria_u_p;
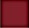
 Proteobacteria;
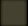
 *Cyanobacteria*;
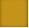
 *Gemmatimonadetes*;
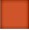
 *Spirochaetae*;
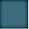
 Peregrinibacteria;
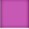
 *Chloroflexi*;
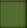
 Patescibacteria;
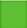
 *Planctomycetes*;
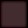
 *Acidobacteria*;
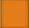
 Ws1;
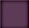
 Tenericutes;
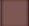
 Deferribacteres;
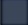
 others.


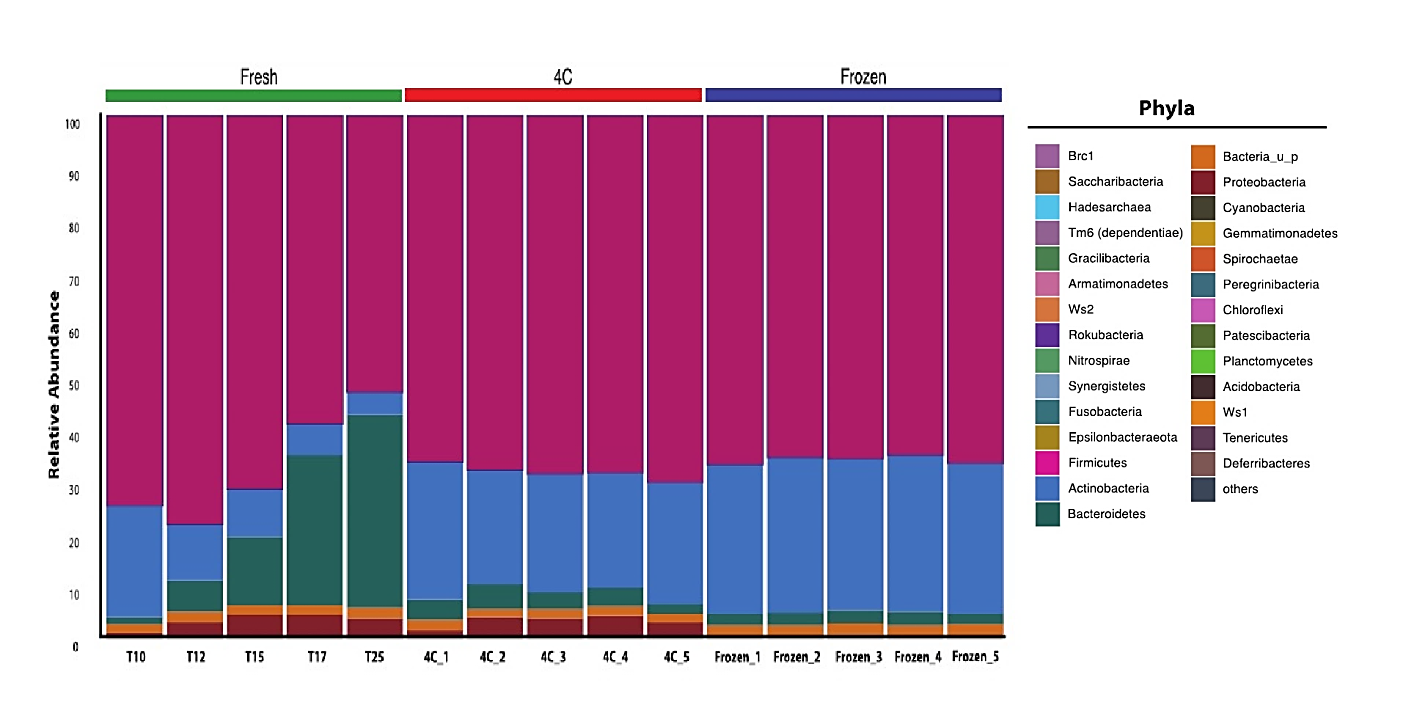


**Figure 6**. *Firmicutes* to *Bacteroidetes* ratio for aliquots analyzed from three different sample processing protocols. The ratio of *Firmicutes*:*Bacteroidetes* (F:B) was plotted as a side-by-side bar graph to illustrate the variations in F:B ratio for each sample processing protocol and sampling time.


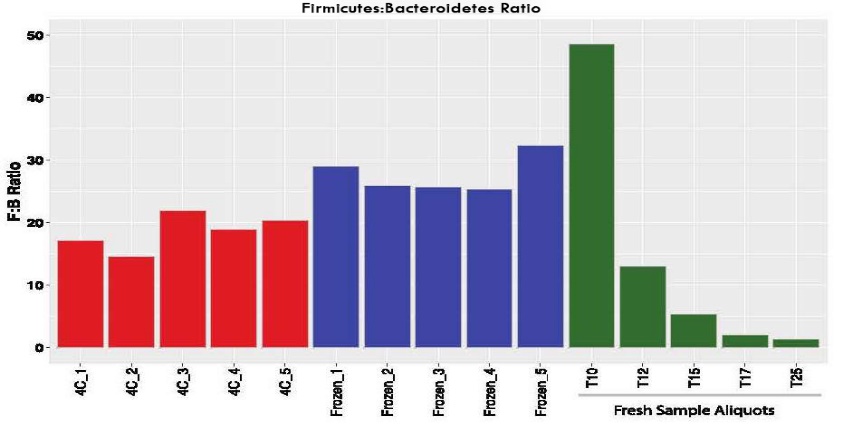


**Figure 7**. PCoA analysis of for aliquots analyzed from three different sample processing protocols. The PCoA plot shows distinct clustering of the stool samples based on the sample processing protocol used. The legend indicates the 3 different sampling processing protocols used in this study. The plot illustrates the coordinates for each individual sample. **Note**:
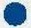
 Fresh;
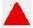
 4ºC
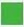
 Frozen.


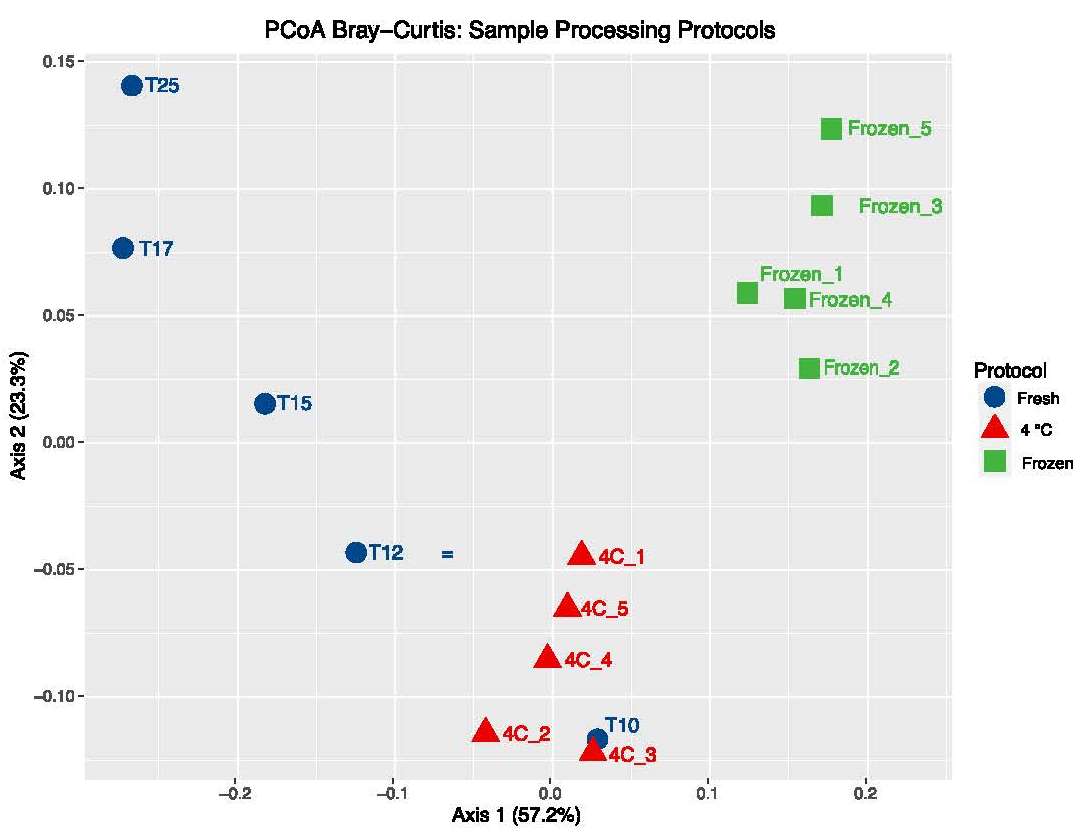

Supplement: Figures RRJMB| Volume 12 | Issue 1|March, 2023 [file NIHMS1904187-supplement-Figures_RRJMB__Volume_12___Issue_1_March__2023.docx]
